# Environmental Factors Influencing Adoption of Canadian Guidelines on Smoking Cessation in Dental Healthcare Settings in Quebec: A Qualitative Study of Dentists’ Perspectives

**DOI:** 10.3390/dj4040040

**Published:** 2016-11-03

**Authors:** Pascaline Kengne Talla, Marie-Pierre Gagnon, Aimée Dawson

**Affiliations:** 1 Centre de Recherche du CHU de Québec (CRCHUQ)—Hôpital St-François d’Assise, Québec, QC G1L 3L5, Canada; 2 Faculty of Nursing, Laval University, Quebec, QC G1L 3L5, Canada; 3 Faculty of Dental Medicine, Laval University, Quebec, QC G1L 3L5, Canada; aimee.dawson@fmd.ulaval.ca

**Keywords:** clinical practices guidelines, smoking cessation, dentists

## Abstract

**Background:** This study aimed to understand dentists’ perspective of the environmental determinants which positively or negatively influence the implementation of Canadian smoking cessation clinical practice guidelines (5As: Ask-Advise-Assess-Assist-Arrange) in private dental clinics in Quebec. **Methods:** This study used a qualitative design and an integrative conceptual framework composed of three theoretical perspectives. Data collection was conducted in individual semi-directed interviews with 20 private dentists lasting between 35 and 45 min. The audio-recorded data were transcribed verbatim, followed by a directed content analysis. **Results:** Some of the barriers identified to counselling in smoking cessation were lack of time, patient attitude, lack of prescription of nicotine replacement therapies, lack of reimbursement, and the lack of training of the dental team. Enablers cited by participants were the style of dentist’s leadership, the availability of community, human and material resources, the perception of counselling as a professional duty, and the culture of dental medicine. In addition to these variables, dentists’ attitude and behaviour were affected by different organisations giving initial or continual training to dentists, governmental policies, and the compatibility of Canadian smoking cessation guidelines with the practice of dentistry. **Conclusion:** Our findings will inform the development of smoking cessation interventions in dental healthcare settings.

## 1. Introduction

Variation in clinical practice undoubtedly affects the quality of services and care received by patients [1]. To mitigate and promote the standardisation of supply of services and care, clinical practice guidelines have been proposed [2,3] to support the clinical judgement of health professionals [4]. The Canadian Action Network for the Advancement, Dissemination and Adoption of Practices in Treatment of Smoking (CANADAPTT), established in 2008, produced the Canadian Smoking Cessation Clinical Guideline (CSCCG) based on clinical experience in the Canadian context [4]. Five major statements are offered to healthcare professionals: Ask-Advise-Assess-Assist-Arrange (5As). Overall, it is recommended to healthcare professionals that they (1) inquire about the smoking status of every patient; (2) advise smokers to quit smoking; (3) assess their willingness to start treatment for quitting; (4) offer assistance to smokers according to their degree of motivation by validated means and ensure smokers attend follow-up sessions to avoid relapses; and (5) also refer smokers to specialized resources.

In fact, tobacco is a risk factor for many oral and systemic diseases and conditions with numerous adverse effects on oral health including a dose-response relationship between smoking exposure and periodontal morbidity [5], aesthetic considerations, and alteration of local physiology and microbiology [6]. In this context, dental visits can be an opportunity to promote cessation counselling through brief interventions [7]. As healthcare professionals, oral health providers are viewed as a credible and reliable source of health information [8] and are recommended to promote smoking cessation in their clinical practice [9].

The majority of studies on the adoption of smoking cessation guidelines in dental clinics focus on individual attributes [10,11,12,13,14]. Not surprisingly, like to other areas of healthcare [15,16], literature on environmental dimensions relating to the provision of smoking cessation services is lacking in the field of dentistry [17,18,19]. Furthermore, the use of explicit theoretical underpinning has often been lacking in previous studies [17,20]. However, we must recognize that the adoption of practice guidelines can sometimes require a change in behaviour of the dental health professional [6,21,22]. Even for professionals who adopt evidence, the results are often mixed and the adoption process is incomplete [23,24,25,26,27]. There remains a limit in the understanding of factors affecting the use of research results in dentistry [3,15]. It has been shown that the use of evidence in healthcare and service organisations is influenced by multiple factors [15,28]. In this context, McGlone et al. (2001) [15] emphasized the relevance of performing research on changing professional practice in the dental field in order to understand the mechanisms underlying the adoption of research findings into clinical routine.

This study is part of a doctoral project aiming to explore the individual and environmental factors that affect the implementation of the Canadian smoking cessation guidelines in private dental clinics in Quebec. This article focuses on environmental determinants, as individual determinants will be reported elsewhere. This study aims to understand, from dentists’ perspectives, the facilitating factors and barriers affecting the implementation of CSCCG to support adult smokers in quitting. Our assumption is that a small number of dentists have an evidence-based approach to brief counselling and that their behaviours are influenced by multiple and various environmental determinants.

## 2. Materials and Methods

### 2.1. Conceptual Framework

**Figure 1:**
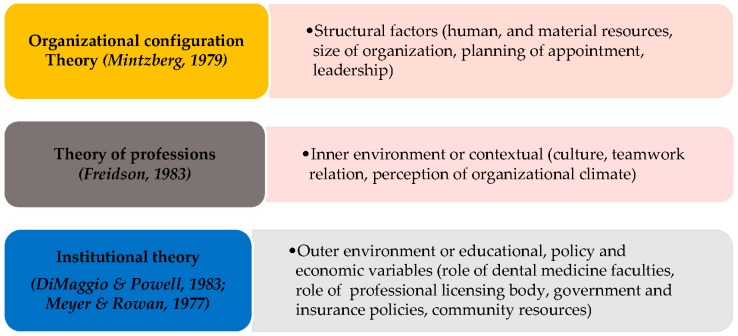
Integrative theoretical model for the study on environmental factors in dental practice settings.

The conceptual framework on environmental determinants used in this study is a combination of constructs from three theoretical foundations (Figure 1).

### 2.2. Methods

#### 2.2.1. Ethical Considerations

This project received ethical approval from the Research Ethics Committee of Laval University (No. 2014/111 R-1). No financial incentive or professional fee was offered to participants. Participants were informed that their participation was voluntary and confidential. The consent form was emailed to them before their participation in the study. A participant’s consent was implicit to their participation to the study.

#### 2.2.2. Study Design

This is a qualitative study based on a convenience sample of dentists practicing in private dental clinics in Quebec. This study is exploratory in nature and it is, to our knowledge, the first study in Quebec on the understanding of the environmental variables that influence the adoption of CSCCG in private dental clinics. Only a qualitative design aims at any in-depth understanding of a new phenomenon and, in this case, to capture the common elements that emanate from individual experiences and the beliefs and perceptions of dentists practicing in the private sector.

#### 2.2.3. Selection of Participants

The inclusion criteria for participating in the study were being a dentist member of the Professional Order of Dentists of Quebec (PODQ), engaging in clinical practice exclusively in the private sector or a mixed practice (private and public), and interacting with patients. The sampling frame was the list of dentists working in Quebec province. This list of names and phone numbers was obtained from the PODQ and was kept confidential by the student researcher. The selection of dentists was based on a probabilistic purposive approach. We were driven by the desire to have participants from different geographical regions of Quebec.

#### 2.2.4. Process of Data Collection

The interview guide constructed from the components of the three conceptual frameworks listed above [29,30,31,32], included a section on structural factors, a question on contextual or organizational factors, and a final section on environmental determinants external to the dental clinic context (see Supplementary Material). The interview guide was validated by five dentists and experts in the field of research in healthcare organizations. These experts commented on the relevance and clarity of the questions, and the ability to cover all aspects of the study to meet the purpose of the study.

Initially, telephone contact was made with the secretaries in dental clinics to explain the purpose of the study and its privacy, and to inquire about the key informant at the dental clinic. A member of the research team conducted the interviews, which took place either in a room of the dental clinic or via the telephone. These semi-structured individual interviews were mainly conducted by telephone. The advantages of individual interviews are that they were personalized and confidential. Thus, participants had the opportunity to express themselves without fear of judgement from their peers [33,34]. The interviews were recorded with the permission of the participants. Notes were sometimes taken to complete the records.

#### 2.2.5. Data Analysis

Direct content analysis approach proposed by Hsieh and Shannon (2005) [35] was adopted to analyse the data. We used a deductive–inductive approach in our analyses. The deductive approach involves a more structured process compared to an inductive approach [35,36]. The recorded interviews were transcribed verbatim. After several readings, transcribed and anonymised data were imported to the Atlas.ti software [37]. Data were initially coded according to the theoretical framework, and general themes subsequently emerged. Four interviews were analysed independently by the student-researcher (PKT) and an associated researcher. In case of disagreement, a consensus was sought. The student researcher codified the remaining 16 interviews. During the analysis, it appeared that some important elements related to the implementation of the Canadian Smoking Cessation Guideline were difficult to interpret based on our conceptual framework. Thus, using an inductive approach, the student-researcher identified emerging themes that could not be included in the existing categories. This process was validated by the other authors (MPG and AD), who are the thesis supervisors.

## 3. Results

The first part will focus on the description of the sample in this study. Successively, emerging topics will be addressed, and this section will conclude with the presentation of results based on the questions in the interview guide. The rationale of our approach was based on our judgement of relevant information. We selected quotes that were poignant and/or most representative of the research findings. We searched to identify emerging themes within the research project or to group various elements into a theme. Also, we wanted to present outlying or negative/deviant cases that did not fit with the central interpretation. For instance, if one fact was reported by one dentist that was contrary to others participants’ reports, we thought it was important to mention it.

### 3.1. Participants and Dental Clinics Characteristics

The results of the descriptive analysis are presented in Table 1. A total of 20 dentists participated in individual interviews, each lasting an average of 40 min. The sample consisted of 11 men. The practical experience of participants varied from 1.5 years to 48 years, with an average of 18.7 years (SD = 14.2). Nearly two-thirds of participants reported never having had knowledge of the CSCCG before this study (*n* = 13). A similar number of dentists surveyed were dental clinic owners (*n* = 14).

More than three quarters of dental clinics were group practices (*n* = 17). According to the regional grouping based on the economic classification of regions of the Ministry of Economy, Innovation and Exports [38], half of participating dental clinics were located in urban regions (*n* = 10).

The dental teams were diverse in numbers and type of dental professionals. According to the data collected, the number of dentists in the participating dental clinics, including dental specialists, varied from 1 to 6 people, with an average of 3.1 dentists (SD = 1.6). If we consider only the dentists in the group practices, their average number was 3.5 dentists per practice. The average number of dental hygienists was higher, with a mean of 4.7 hygienists per practice (SD = 3.8).

### 3.2. Results from the Inductive Analysis Approach

#### 3.2.1. Patient

Participants brought up “the patient” as a major factor in the dentist’s behaviour in implementation of CSCCG in private dental clinics. The participants made reference either to the patient’s oral health status and the patient’s accountability, or by expressing the importance of responsiveness and openness on the practitioner’s behaviour. Indeed, the patient’s attitude can stimulate the dentist to offer smoking cessation services.

“*If the initiative comes from the patient who has seen a poster or a TV spot in the waiting room, this action can stimulate the practitioner to help smoker for quitting*.”

A dentist noted the relevance of considering the social context of the smoker in the adoption of CSCCG in dental clinics.

“*There are places where tobacco use is a generational issue and disadvantaged settings where smoking is the pleasure of life. Thus, the dentist must consider these aspects when he wants to support a smoker who wants to quit smoking*.”

#### 3.2.2. Characteristics of Canadian Smoking Cessation Guideline

The participants in this study reported that CSCCG must possess certain characteristics, such as compatibility, to be used in the dental clinic. This aspect can address the duality of dentistry as a healthcare area and a business enterprise.

“*The existence of a specific, clear and concise protocol is a prerequisite for my involvement, as well as reimbursement and the possibility of writing a prescription. If I have to be reimbursed for smoking cessation services and I do not have direct directions to follow, this would take me too long and I do not want to spend extra time on this service, we need a protocol*.”

#### 3.2.3. Perception of the Statements of Canadian Smoking Cessation Guidelines

When broadly discussing the statements of CSCCG commonly used in clinic, the participants report that three statements of the CSCCG—namely, “Ask-Advise and Arrange/refer”—were the standard services. The patient is currently referred to physicians and specialists in oral medicine, maxillofacial surgery, and periodontal health.

Expressions such as *explain the benefits of quitting, educate the patient about the consequences of smoking related to oral health, encourage the patient to engage in smoking cessation,* and *help smoker by giving him advice—guide, direct the smoker to the appropriate professionals* refer to concepts often mentioned by study participants about the type of intervention for quitting smoking. Thus, other statements of CSCCG such as “Assess-Assist” are not perceived as feasible within the dental clinic context.

“*The dentist does not have the role to monitor the patient during the smoking cessation process, just to raise awareness about the consequences of smoking*.”

#### 3.2.4. Structural Factors that Influencing the Implementation of CSCCG in Private Dental Clinics in Connection with Mintzberg’s Organizational Configuration Theory

##### Receptive Working Environment

Participating dentists reported that structural factors have an impact on the provision of smoking cessation services by emphasizing the availability of various kinds of human and/or material resources. According to respondents, having human resources depends on the size of the dental team, but also having qualified resources and training in the provision of smoking cessation services. The size of the dental team, referring to the number of dentists or the number of other oral professionals within the dental clinic, seems to be linked with the type of practice.

“*Practice groups with support staff have the opportunity to offer smoking cessation services. If individuals are mandated to do certain tasks, it provides the time to the help the patient and the opportunity for dentists to take more time with the patient*.”

In terms of material resources, more than half of participants reported that these tools promote smoking cessation within private dental clinics (*n* = 12). Study dentists cited pamphlets, posters, TV commercials, or reminder stickers. Among these support systems, pamphlets are the most used.

“*The pamphlet is the most used as a promotional tool, as it helps to start the conversation and the patient can view it in the waiting room in the dental clinic*.”

##### Leadership in Clinical and Administrative Decisions

Participants expressed the leadership of the dentist as an important issue in implementation of CSCCG. For participants, this concept includes expertise, professionalism, training, personal philosophy of practice oriented towards a global approach to health, and especially his autonomy in clinical decisions. Nevertheless, this observation is not always applicable to dentists who have newly entered the job market.

“*Every dentist is responsible for his treatment plan. He is responsible for his patients. We can work with a colleague for some treatment plans, depending on the case, working either in collaboration or consultation*.”

“*Young graduates will move towards a group practice, working as associate dentists. In this case, they are more likely to adhere to the philosophy of the owner of the dental clinic and to follow their guidelines*.”

Another point described in the leadership of the dentist, especially for owner dentists, involves administrative decisions, which include not exclusively planning consulting time, staff hiring, and work schedules. Participants are almost unanimous about the impact of the daily or weekly volume of patients on assistance to smokers in quitting smoking in private dental clinics.

“*If a clinic is too loaded with appointments between patients every 30 min, it is certain that the workload will negatively influence the support provided to smokers*.”

#### 3.2.5. Factors Related to the Internal Environment Influencing the Implementation of CSCCG in Private Dental Clinics in Connection with Friedson’s Theory of Professions

##### Professional Duty in the Healthcare Context

The majority of study participants were aligned on professional and social roles of dentists according to their expertise and their social contract. However, participants noted the limitations in these professional duties. As an example, participants commonly raised the lack of skills, the lack of training, and the lack of beliefs in the effectiveness of their interventions. Another barrier identified as influencing the implementation behaviour of CSCCG is the bipolarity of dentistry, such as it being both a medical profession and business enterprise.

“*It is difficult for us to survive as a liberal profession in a public context, to find a balance between a profitable practice and the patient’s needs; we are facing a problem of time and efficiency. Thus, the financial issue over the patient’s health is an important issue*.”

Besides the role of the general dentist, participants brought up the role of dental specialists in supporting smokers. Several dental specialists are perceived as being more in touch with smokers, due to their scope of practice, the role of tobacco on the prognosis of their interventions, and the regularity in which they see smokers. Among these specialists, participants cited specialists in periodontal health, oral medicine, and maxillofacial surgery.

##### Culture

The practice of dentistry is seen as teamwork by the majority of participants in this study, whatever the type of practice. The majority of participants reported the positive impact of the close working relationship of the dentist and his or her staff (90% of participants), and the effect on patients’ satisfaction.

“*Given this consistency in the messages internally, the patient may be aware of the seriousness of the problem and be reassured of the support if he wants to quit*.”

In this teamwork, the dentist commonly shares a role with the dental hygienist. It is almost unanimously recognised that the dental hygienist plays a pivotal role in the instruction of the patient on his or her health. However, participants believe that the dentist’s contribution is also important.

“*The dental hygienist is the first person to see the smokers, they are the first contact. However, I see no harm in the dentist adding support to the dental hygienist*.”

#### 3.3.6. Factors Related to the Outer Environment in the Implementation of CSCCG in Private Dental Clinics in Connection with Institutional Theory

##### Complementary Training Organisations: Dental Faculties and the Professional Licensing Body of Dentists in Quebec

When asked about the external variables which are likely to influence dentists in the adoption of CSCCG, participants highlighted the catalytic role of the dental schools for future dentists and interest in extending their scope of practice through the possibility of prescribing nicotine replacement therapy. This extension of practice could be achieved by the professional licensing body in Quebec. The role of academia among dentists refers to education, awareness, encouraging the adoption of healthy lifestyles, and promoting the status of dentist as an expert authority on oral health. Also, some participants stressed the dose-response relationship between the future dentist’s awareness and the probability of getting more involved in the support for cessation of smoking.

“*A participant mentioned the relevance for dental faculties to bring the community health course earlier to the undergraduate, to familiarize future dentists with an overall health approach, to give them time to internalize it, and develop the skills and abilities to effectively intervene with smokers*.”

##### Government Policies

Participants noted the actions that have been performed to date in terms of government policies in the awareness of smoking by the population, and the recommendations for dentists as health care professionals. In light of these actions, dentists emphasise that they have a professional duty to help.

“*Dentists need to strengthen actions that have been undertaken by the government, the information needs to be conveyed that is out there. Government activities are incentives for practitioners. It is a way to make them understand that the ball is thrown, the train is leaving the station and that everyone should hop on*.”

##### Economic Issues

Time constraint was expressed as the main factor likely to affect the support for quitting smoking in private dental clinics by almost all dentists surveyed. The time devoted to supporting smoking cessation is seen both as part of the time of patient care, and as additional working time. These two ways of thinking have also generated mixed views on the coverage of smoking cessation services by insurance policies, and especially its impact on dentists’ attitudes and behaviour in private practice.

“*You cannot ask for money for all the information and advice given to patients. It’s like checking tissue during an examination for oral cancer, as well as talking about alcohol with patients*.”

“*Yes, it might be recognition of their time that could encourage dentists to become more involved and that their time is not volunteered, this can make a difference if there is a reasonable fee*.”

A last participant adopts a perspective of reducing social inequalities related to smoking. He states: “*In view where there would have an opportunity for reimbursement of smoking cessation services received in the dental clinic, it must be done via the public health insurance plan of Quebec (RAMQ) and not private insurance.*”

## 4. Discussion

This study used a descriptive qualitative design to understand private dentists’ perspectives on the environmental variables that may affect the implementation of Canadian Smoking Cessation Clinical Guidelines (CSCCG) in Quebec. We assumed that the decision to offer tobacco cessation services in a private clinic is influenced by a multitude of internal and external factors which themselves influence each other. Thus, due to the limitations of a single theoretical approach, the use of an open and inclusive model was preferred. Based on semi-structured interviews with a convenience sample of geographically diverse participants, a direct content analysis of collected data was performed.

Results from this study confirm our assumption. The general consensus arising from this study is the lack of knowledge of CSCCG by Quebec dentists. Despite their willingness to contribute to support smoking cessation and to participate in this study, one-third of the surveyed dentists had never heard of CSCCG before this study. Moreover, even among those dentists who had heard of CSCCG, they were not familiar with their content.

Another finding from the analysis is that the logic that governs the delivery of smoking cessation services in private dental clinics is more patient-oriented than directed towards the standards of best practices. Dentists give a central place to their patients, and their interventions underlie the initiative of the latter. Note that if the CSCCG should be used, these guidelines should meet compatibility standards with the duality that prevails in the practice of dentistry and its features, especially as a medical profession and a business enterprise. Although dentists surveyed appear to be in an excellent position and have the willingness to offer the smoking cessation services as part of “usual” care, they see barriers and facilitating factors, both structurally and related to the internal and external environment.

The culture of dentistry involves values and professionalism. In addition to social values, the practice of dentistry is characterised by various theoretical, economic, and political values [39] placing this field at the intersection between a biomedical model and an economic perspective [40]. This duality will affect the supply of health services in many aspects, namely, structural, ideological, and economic.

On the structural side, the two components of professionalism as defined by Zijistra (2013) [41], normative and ideological knowledge, are expressed in this study. The normative component of professionalism refers to dentist’s leadership and its monopoly on the provision of smoking cessation services in the dental clinic [42,43,44]. Accountability of the dentist is seen through the control of certain mandates assigned to other oral professionals in the dental clinic, the need to strengthen the work done by the dental hygienist, and leadership in teamwork. This perception of supervision of dental hygienists’ work to support smoking cessation is recognized in the literature [45]. For Friedson (1983) [32], this position would result from the existing interaction between power and knowledge in medical fields.

The dual function of several dentists as both manager and owner probably affects the adoption of innovations, according to studies on organisations quoted by Minztberg [46] (p. 301). Thus, the dentist’s vision of his or her practice will predispose the clinical environment to smoking cessation interventions through the planning of patient consultation time, the time between appointments, the estimated daily volume of patients, and the type of practice (solo or group). In this study, the concept of the type of practice is equated with the concept of organisation size, such as the literature on the relationship between the size of an organisational structure and the adoption of innovations [46]. In addition to planning services, dentists intervene in the modification of the physical environment in their dental clinic. Indeed, these material resources appear to be related to the availability of tobacco control tools within the dental clinic, which can be displayed or distributed to patients. Thus, the creation of a tobacco-free dental clinic, through the use of the resources within the organisation, is an opportunity whose effect is two-dimensional. The patient intuits the dental clinic values from the waiting room and can initiate the conversation about smoking cessation. In addition to patient prompts, these tools can be reminders for the teamwork to intervene with smokers [47]. However, some participants were sceptical of the existence and use of smoking cessation tools in dental clinics. Ultimately, if dentists feel more comfortable talking directly with their patients, this does not exclude the availability of additional materials in the waiting rooms. This result reinforces other studies’ findings on the context and the implementation of best practices [48,49]. It is shown that the creation of a healthy environment is a preventive measure and a health strategy [50].

The ideological component of dental professionalism [40] is characterised by the recognition of the role of the dentist in supporting smokers. As health professionals, dentists fully recognise their professional duty to provide support to smokers in private practice. This result corroborates others in the literature on the recognition of professional responsibility of dentists in supporting smokers [13,25,51,52,53]. This observation on the effect of symbolic and cultural aspects on the adoption of innovations is consistent with the results of studies in the field of organization of services [46,54,55,56]. However, participants reported limitations in their roles—for instance, sharing of functions with the dental hygienist or other healthcare professionals—and more often mentioned the family physician.

Regarding the economic component, the surveyed dentists have expressed the impact of the lack of coverage of smoking cessation services on support for smoking cessation in private dental clinics. This observation is consistent with results in the literature on the lack of financial incentives on dentists’ attitude and behaviour [19,47,57]. Lack of reimbursement is likely to affect the economic benefit received by dentists to support interventions for quitting. These findings confirm and support Rogers [58], who showed that the adoption of an innovation depends on its social or economic benefit, or both. However, it should be noted that dentists were divided on the reimbursement of support to smokers in dental clinics. On the issue of coverage of smoking cessation practices, in United States, insurers indicated a need for more evidence of clinical and cost-effectiveness interventions before the change of the current reimbursement structure for these services [23].

Professionalism is taught as part of the dental curricula, but also during continuing education training [40]. Participants in this study reported that dental faculties will contribute to the development of professionalism by providing necessary and appropriate tools to future dentists to increase their personal effectiveness. This assertion is largely consistent with the results in the literature. For example, it is reported that the role of dental schools is in building values, but also the skills, attitudes, and behaviours that will mark the future employment of graduates in dentistry [40]. Moreover, Albert et al. (2004) [59] noted the limitation and non-systematisation of courses on smoking cessation in dental schools, and efforts too often directed to the consequences of smoking. Similarly, one of the dentists emphasized the delay of a community health course in dentists’ training process. So, this participant mentioned the relevance of placing this course earlier in the program timeline, to familiarise future dentists with an overall health approach, to give them time to internalise it, and develop the skills and abilities to effectively intervene with smokers. One last thing emphasised in this study is building the social exemplary status of dentists. This statement is consistent with the characteristics of a leader [60,61]. In the same vein, Christen (1984) [62] noted that dentists should position themselves as models of health choices for population.

In addition to the groundwork established during the training in dentistry, the role of the provincial licensing body and government policies in promoting the offer of smoking cessation services is reported by the surveyed dentists. In Quebec, some legislative and financial measures were adopted to encourage health professionals in smoking cessation counselling, for instance, the collaboration established in 2004 between the Public Health Institute of Quebec and six health professional orders, reimbursement of nicotine replacement therapy and bupropion through public and private drug insurance plans, and the establishment of 160 smoking cessation centres across the province [26,63] Nevertheless, the participants reported that enhancements involving smoking cessation activities from government and dentists’ provincial licensing body will be interesting. For example, participants suggested strengthening education of the population on the role of the dentist in supporting smoking cessation. It appears that the joint relationship of professional education and public education have an impact on raising mutual awareness and late diagnosis of cancer [64].

### 4.1. Strengths and Limitations

This is the first study, to our knowledge, in Quebec and possibly in Canada that explores the environmental factors that may affect the implementation of CSCCG in private dental clinics. Also, the absence of a regional difference in perceptions of participants in this study, and the diversity of the sample lead us to believe that the results of this study may generally reflect the views of dentists in the province of Quebec. Moreover, the exercise of dentistry is a private practice in most industrialized countries, and even in developing countries; so, it can be assumed that the issues are likely to be the same in many ways, with some exceptions. In this context, the results of this study could be transferred to similar contexts. The internal validity of the study is provided by repeatedly reading verbatim, coding, and verification of codes made independently by two people. Another benefit in this study is the familiarisation of the members of the research team with dentistry.

In terms of limitations, the results remain fragmented because it is only the dentists’ perspective, which does not give a complete portrayal of the perception of the dental team on factors affecting the supply of smoking `cessation services. Secondarily, the voluntary participation of dentists in this study may have influenced the responses. Data are self-reported and interviews were performed by phone, so participants’ responses could be overestimated about some questions. Besides, the quality of a qualitative study is dependent on the skills of the researcher [65,66,67]. The student is a junior researcher in qualitative research, and that experience may have affected the data analysis process. However, discussions were conducted with the supervisors and another researcher to validate the data analysis structure. Although the interviewer took time to explain the purpose of the study, participants may have answered questions by ignoring this information and keeping in mind the general idea of offering smoking cessation services.

## 5. Conclusions

The qualitative design of this study sheds light on the diversity of environmental factors, at different levels, which influence the implementation of CSCCG in dental clinics in Quebec. An important result from this study is that only one-third of the surveyed dentists had heard of CSCCG before this study. In this case, strategies must be developed to inform more dentists about these guidelines. In addition to these factors, our findings highlight the duality characterising the exercise of dentistry positions by dentists both as entrepreneurs [68] and health professionals. In this context, the patient has a major role in the supply of tobacco cessation services in private practice, considering all its characteristics such as its health status, its responsiveness, and its social context. These findings will be confirmed or complemented by a quantitative study that is currently underway.

## Figures and Tables

**Table 1:** Participants’ characteristics.

Variables (*n* = 20)	Frequency (*n*)	Range or Percentage (%)	Mean (SD)
Gender			
Female	9	45	
Male	11	55	
Years of experience in the current dental clinic		10 to 40 years	11.7 (11.2)
Years of experience in dentistry		1.5–47 years	18.7 (14.2)
Previous knowledge of Canadian smoking cessation guidelines before this study			
Yes	13	65	
No	7	35	
Owner of dental clinic			
Yes	7	35	
No	13	65	
Number of dentists in participants’ dental clinics		1–6	3.1 (1.6)
Number of dental hygienists in participants’ dental clinics		0–18	4.7 (3.8)
Type of practice			
Solo	3	15	
Group practice	17	85	
Number of patients seen by week			
16–29	6	30	
30–44	4	20	
45 and more	10	50	
Perception of workload			
Heavy	8	40	
Average	12	60	
Having a dental manager or dental coordinator			
Yes No	13 7	65 35	

## Supplementary Materials

The following are available online at http://www.mdpi.com/2304-6767/4/4/40/s1, Interview guide for individual interviews.
