# Supplementary material for: Environmental Factors Influencing Adoption of Canadian Guidelines on Smoking Cessation in Dental Healthcare Settings in Quebec: A Qualitative Study of Dentists’ Perspectives
Source: Dent J (Basel). 2016 Nov 3;4(4):40. doi: 10.3390/dj4040040 (PMC5806960; doi:10.3390/dj4040040)
Supplement: Supplementary file 1 [file dentistry-04-00040-s001.pdf]

dentistry journal

Supplementary Materials

Interview Guide for Individual Interviews

Pascaline Kengne Talla, Marie-Pierre Gagnon and Aimée Dawson

Project's Title: Environmental Factors Influencing Adoption of Canadian Guidelines on Smoking Cessation in Dental Healthcare Settings in Quebec: A Qualitative Study of Dentists' Perspectives

Instructions

1. Your participation in this study is anonymous and your answers are confidential.
2. Your participation at this interview implies your consent
3. There are no right or wrong answers.

The goal of this interview is to understand private dentists' perspective of the environmental determinants which positively or negatively influence the counselling of smoking cessation with adult smokers in Quebec (new and existing patients).

What are the Canadian guidelines on smoking cessation?

The guidelines are a tool used by health professionals including dentists to help smokers quit, consisting of five sequential statements that health professionals are invited to use:

Ask: Health care providers should ask about tobacco use status of patients/clients on a regular basis.
Advise: Health care providers should clearly advise patients/clients to quit.
Assess: Health care providers should assess the willingness of patients/clients to begin treatment to achieve abstinence (quit attempts).
Assist: Health care providers should offer assistance to every tobacco user who expresses a willingness to begin treatment to quit via minimal interventions of 1 to 3 min incorporating counselling and advice.
Arrange: Health care providers should conduct regular follow-ups to assess response, provide support and modify treatment; as necessary; and, are encouraged to refer patients/clients to existing relevant resources (examples in Quebec are smoking cessation centres, the program "j'Arrrête j'y gagne", and family physicians).

The behaviour of interest for today's discussion is «using Canadian smoking cessation guidelines (CSCCG) defined as applying at least one of the five statements in his or her daily practice». I'm going to keep emphasizing this throughout the interview.

Sometimes, I may ask for clarification of certain responses during the interview using probes such as: *"Would you explain that: 'Take me through the experience'; 'What skills are required to do so?'; 'What do you mean by…?'*

1. To what extent do structural factors influence the implementation of smoking cessation guidelines in private dental clinic?

Prompts: process of clinic and administrative decisions, number of dentists and others personal in the dental clinic, planning appointment, type of appointment, frequency of appointment, date of acquisition of this dental clinic by the current owner, kind of dental specialists.

2. What aspects of your inner environment have an impact on the implementation of smoking cessation guidelines in private dental clinic?

Prompt: role of dentists, teamwork, delegation of tasks, physical resources, and organisation of working climate

3. To what extent do outer environment factors influence the implementation of smoking cessation guidelines in private dental clinic?

Prompts: insurance policy, government policies, dental medicine faculties, professional licensing body of Quebec dentists, community resources, role of prescription of nicotine therapies replacement…

4. Sociodemographic variables

a. What is your year of birth? ___________________________________
b. What is your gender? Male ☐ Female ☐
c. How many years of clinical practice experience do you have as a dentist? ____________
d. How many years of clinical practice experience do you have as a dentist in your current dental clinic? ____________
e. In what region is your principle place of practice of dentistry? ____________
f. Are you the owner of this dental office? Yes ☐ No ☐
g. What is the type of practice of your primary workplace? Solo practice ☐
Group practice ☐
h. What is your employment status?
Solo dentist practice owner ☐ Associate dentist ☐
Dentist engaged as a renter of chair(s) in another dentist's practice ☐
i. How heavy do you feel the workload is in your dental clinic is?
Heavy ☐ Average ☐ Low ☐
j. What is the average number of patients seen weekly in this dental clinic (by you and for all dentists)? Less than 15 ☐ 16–29 ☐ 30–44 ☐ 45 and more ☐
k. Before this study, had you already heard about Canadian smoking cessation guidelines?
Yes ☐ No ☐
Those are all the questions I had for you today. Has anything else occurred to you about this topic that I haven't asked about?

Thanks you for your time and your collaboration.
